# Contributions of the psychology of mathematical cognition in early childhood education using apps

**DOI:** 10.3389/fpsyg.2022.913970

**Published:** 2022-09-06

**Authors:** Carlos Mera, Cándida Delgado, Estíbaliz Aragón, Inmaculada Menacho, María Del Carmen Canto, José I. Navarro

**Affiliations:** Department of Psychology, University of Cádiz, Cádiz, Spain

**Keywords:** digital learning games, app, mathematical cognition, magnitudes comparison, numerical estimation, early education

## Abstract

Educational interventions are necessary to develop mathematical competence at early ages and prevent widespread mathematics learning failure in the education system as indicated by the results of European reports. Numerous studies agree that domain-specific predictors related to mathematics are symbolic and non-symbolic magnitude comparison, as well as, number line estimation. The goal of this study was to design 4 digital learning app games to train specific cognitive bases of mathematical learning in order to create resources and promote the use of these technologies in the educational community and to promote effective scientific transfer and increase the research visibility. This study involved 193 preschoolers aged 57–79 months. A quasi-experimental design was carried out with 3 groups created after scores were obtained in a standardised mathematical competence assessment test, i.e., low-performance group (*N* = 49), high-performance group (*N* = 21), and control group (*N* = 123). The results show that training with the 4 digital learning app games focusing on magnitude, subitizing, number facts, and estimation tasks improved the numerical skills of the experimental groups, compared to the control group. The implications of the study were, on the one hand, provided verified technological tools for teaching early mathematical competence. On the other hand, this study supports other studies on the importance of cognitive precursors in mathematics performance.

## Introduction

Currently, international standardised tests of mathematics achievement do not show encouraging data for Spanish students (Ministerio de Educación y Formación Profesional, 2019; OECD, 2019). Student performance is below average compared to other OECD countries. Therefore, the widespread failure in mathematics learning in our education system has aroused the interest of the scientific community (European Commission, 2020).

The onset of formal learning in the logical-mathematical area occurs at ~5 years of age. According to some authors, this is the age when the first signs of risk learning difficulties appear (Wong and Chan, 2019; Zhang et al., 2020). Numeracy skills in early childhood are the building blocks for the later successful development of mathematical competence (Passolunghi and Lanfranchi, 2012; Hornung et al., 2014). In this sense, it is necessary to consider the predictive role of cognitive processes linked to mathematical achievement at an early age (Zhang et al., 2020). These processes may also represent a critical feature in the detection of students at risk of mathematics learning difficulties (MLD) in later grades (Barnes and Marks, 2020).

The basic numerical skills that support the development of mathematical skills between 5 and 8 years of age focus on the following four main factors (Aunio and Räsänen, 2016): (1) symbolic and non-symbolic number magnitude; (2) understanding of mathematical relationships (logical-relational principle, arithmetic principles, symbols of arithmetic operations, and place value system and base-ten); (3) counting skills (knowledge of number symbols, number word sequence, enumeration with concrete objects); and (4) basic skills in arithmetic (arithmetic combinations, addition, and subtraction skills with number symbols).

In mathematics learning, there are a number of variables that predict performance, and these are usually grouped into two categories, namely, general domain and specific domain (Passolunghi et al., 2015; Ramani et al., 2017). General-domain predictors refer to higher-order cognitive variables such as working memory and processing speed (Fritz et al., 2019), whereas specific-domain predictors contribute to the performance of a particular school skill. There are several domain-specific predictors related to mathematical competence (Aragón et al., 2016), including *magnitude comparison* (Matejko and Ansari, 2016; Xenidou-Dervou et al., 2017) and *numerical estimation* (Reynvoet et al., 2016; Friso-van den Bos et al., 2018).

*Magnitude comparison* is defined as the sensitivity to distinguish numerical quantities. This is an important skill during the early stages of education (De Smedt et al., 2013; Toll et al., 2015). Numerical quantities can be represented symbolically (Arabic digits) or as non-symbolic quantities (a set of dots). Either way, the discrimination of non-symbolic quantities is a predictor of early numeracy skills (Soto-Calvo et al., 2015; Cueli et al., 2019b). In symbolic representation, it is necessary to have the ability to make a correct and immediate identification of each of the numerical symbols represented. They must then contrast the quantities and decide if the number is larger or smaller (Merkley and Ansari, 2016).

Many authors place the representation of non-symbolic magnitude as the predecessor of symbolic magnitude. Some studies show that 5-year-olds perform better on non-symbolic comparisons than on symbolic comparisons (Matejko and Ansari, 2016; Canto-López et al., 2019). In contrast, this difference in performance seems to disappear at age 6. From this age onwards, children tend to achieve similar results in symbolic and non-symbolic quantities. However, as they learn more about the symbolic representation system, the difference with respect to non-symbolic representation narrows (Li et al., 2018). Nevertheless, there are still many scientific questions in this regard, as the data are inconclusive.

*Number line estimation* is another domain-specific cognitive skill that is closely related to the approximate number system (ANS) (De Hevia, 2016; Reynvoet et al., 2016; Zhu et al., 2017). In a typical number line estimation task, children are asked to indicate the position of an Arabic numeral on an empty number line. The line indicates the number 0 on the far left and a larger number (usually 10, 100, or 1,000) on the far right. In the first test, a concrete number is given for the child to estimate the position corresponding to each requested number on the line (number-position). However, the same estimation task can be performed by providing a line with the same characteristics as the previous one but marking a specific position on the line with another small line in a perpendicular position. In this way, the participant has to indicate the number that corresponds to that particular position (position-number) (Siegler and Opfer, 2003).

Number line estimation seems to be important for learning mathematics. In fact, the basis of numerical cognition is considered an innate representation of numerical magnitude as a mental number line (Dehaene, 2003, 2011). Recent studies have shown that this domain-specific cognitive task has a high predictive value for mathematical achievement (Schneider et al., 2017; Cerda et al., 2018; Núñez-Peña et al., 2019).

Although results are not yet conclusive, training the ability to estimate magnitude on the number line may be useful in gaining adequate access to symbolic numbers and their relationship to magnitude. This suggests that the number line can be a powerful representational tool for strengthening connexions between symbols and the quantities they represent (Booth and Siegler, 2008). These cognitive processes underlying children's responses in the estimation task correlate with mathematics achievement even at later educational stages (Geary et al., 2013; Schneider et al., 2018).

Therefore, training these variables considered predictors with digital technology can bring academic benefits (Re et al., 2020). This study contributes to the research on mathematics app use with children aged 4 and 5 years and specifically touch-screen apps that contain digital learning games.

Digital learning games focus on the design of apps or videogames that incorporate learning models and educational content to enhance learning (Prensky, 2003). Digital technologies, such as a *tablet* or *computer applications*, support teachers and offer certain advantages for student learning. In this sense, it is necessary that the software is well-designed and its content based on the child's stage of development (Kucirkova et al., 2014; Hubber et al., 2016).

Several authors consider that technologies, compared to traditional resources of similar structure, can contribute to improving learning (Wouters et al., 2013; Fernández-Abella et al., 2019; Peralbo-Uzquiano et al., 2020). In other words, the tasks, and not the format in which they are presented, would be responsible for the progress.

Numerous benefits have been reported with the app for children aged 4–7 years in need of additional support with learning mathematics. In addition, apps have been shown to help children with poorer short-term memory make greater learning gains than those with higher memory skills (Outhwaite et al., 2017). For this reason, increased time learning mathematics with an educational storey app at home improved children's mathematical skills in primary school (Outhwaite et al., 2019). However, there are some disadvantages to consider. Abuse of apps can lead to the emergence of an addiction to this type of device, as well as promoting individual work and, consequently, social isolation (Bonilla-Barbosa, 2014). Furthermore, excessive use of technology can lead to a decrease in effort in some basic school skills for learning, such as writing (Graham et al., 2013).

There are educational technology games that fit the criteria of the early years' mathematics curriculum (Schacter et al., 2016; Sheehan et al., 2019; Schenke et al., 2020). However, few focus on the cognitive approach to mathematical learning (Aragón et al., 2017; Mera et al., 2019; Peralbo-Uzquiano et al., 2020).

Inside these multiple sceneries, in the last decade, educational psychology has proposed different lines of research related to the cognitive processes linked to mathematical learning at a young age, as well as the possibility of taking advantage of the knowledge available in this relationship to implement programmes with different characteristics. It is in this context that this study is located. In this research, we designed and implemented 4 digital learning app games to train specific cognitive predictors that should influence early mathematical competence in 5-year-old children. There are two objectives as follows: on the one hand, to demonstrate that the teaching of specific cognitive predictors through app games improves mathematical achievement; on the other hand, to empirically verify that the digital educational games designed to provide support in the teaching-learning process of mathematical competence, both in the classroom and at home.

## Materials and methods

### Participants

The total number of participants was 193 preschoolers from middle socio-economic and educational levels of families, whose ages ranged from 57 to 79 months (M = 63.3, *sd* = 3.7). Of them, 107 (55%) were girls, aged between 57 and 70 months (*M* = 63.1, *sd* = 3.4) and 86 (45%) were boys, aged between 57 and 79 months (*M* = 63.4, *sd* = 4). From the total sample of students from 4 schools (two public schools and two subsidised schools), children with special educational needs were excluded, as judged by experts. Percentile scores obtained in TEMA-3 in the pre-intervention phase allowed the construction of the following three groups: high performance in mathematics (percentile higher than 80), low performance (percentile lower than 25), and average performance. Groups were distributed in the following way:

· Low-performance group (*LP group*) consisted of 31 girls (63.3%) and 18 boys (36.7%) aged 57–79 months (*M* = 62.55, *sd* = 4).
· High-performance group (*HP group*) consisted of 9 girls (42.9%) and 12 boys (57.1%) aged 58–70 months (M = 64.6, sd = 3.5).
· Average-performance group (*control group*) consisted of 67 girls (54.5%) and 56 boys (45.5%) aged 57–71 months (*M* = 63.26, *sd* = 3.5).

### Assessment instruments

#### Test of early mathematics ability – third edition

This test is composed of two subtests that focus on the assessment of informal and formal thinking, both in concepts and skills. The informal subtest is composed of tasks aimed at assessing numeracy, quantity comparison, informal calculation, and basic informal concepts. The formal subtest assesses conventions related to the reading and writing of quantities, knowledge of numerical facts, formal calculation, and formal mathematical concepts. The test was administered individually and lasted around 30 min. This time varied according to the age of the pupils. This individually administered test for children between 3 and 8 years of age identifies students with mathematics learning difficulties or at-risk students. It consists of a total of 72 items, presented in order of increasing difficulty, which is administered until the student responds incorrectly to 4 items in a row. Cronbach's alpha was 0.91 (Ginsburg et al., 2007).

#### Symbolic and non-symbolic comparison test

To assess magnitude processing skills, participants were presented with a booklet of symbolic (Arabic digits) and non-symbolic (dots) number pairs and were asked to compare two numerical magnitudes and point (using a pencil) to the larger number within a given time (2 min per modality). The magnitudes vary from 1 to 9 and the side on which the larger magnitude is presented is counterbalanced in all items. For each format, 56 items were presented (56 symbolic pairs and 56 non-symbolic pairs). Cronbach's alpha was 0.86 (Nosworthy et al., 2013).

#### Numerical estimation task

This pencil and paper test assesses number line estimation in its two modalities: *number-position* (a number is presented and the participant must know its position within a straight line), and *position-number*, where a sign is shown on the straight line and the participant must recognise which number it corresponds to. The test consists of 10 items for each modality, corresponding to the following numbers: 2, 4, 7, 8, 11, 13, 16, 17, 18, and 19, randomly presented. The comparison of means was made on the basis of the number of correct answers, with respect to the number requested vs. the number given by the student. For this purpose, the answer was assigned as correct if it did not have an error rate higher than +/- 15% of the number asked for. Cronbach's alpha was 0.80 (Siegler and Booth, 2004).

### Intervention instruments

**Figure 1:**
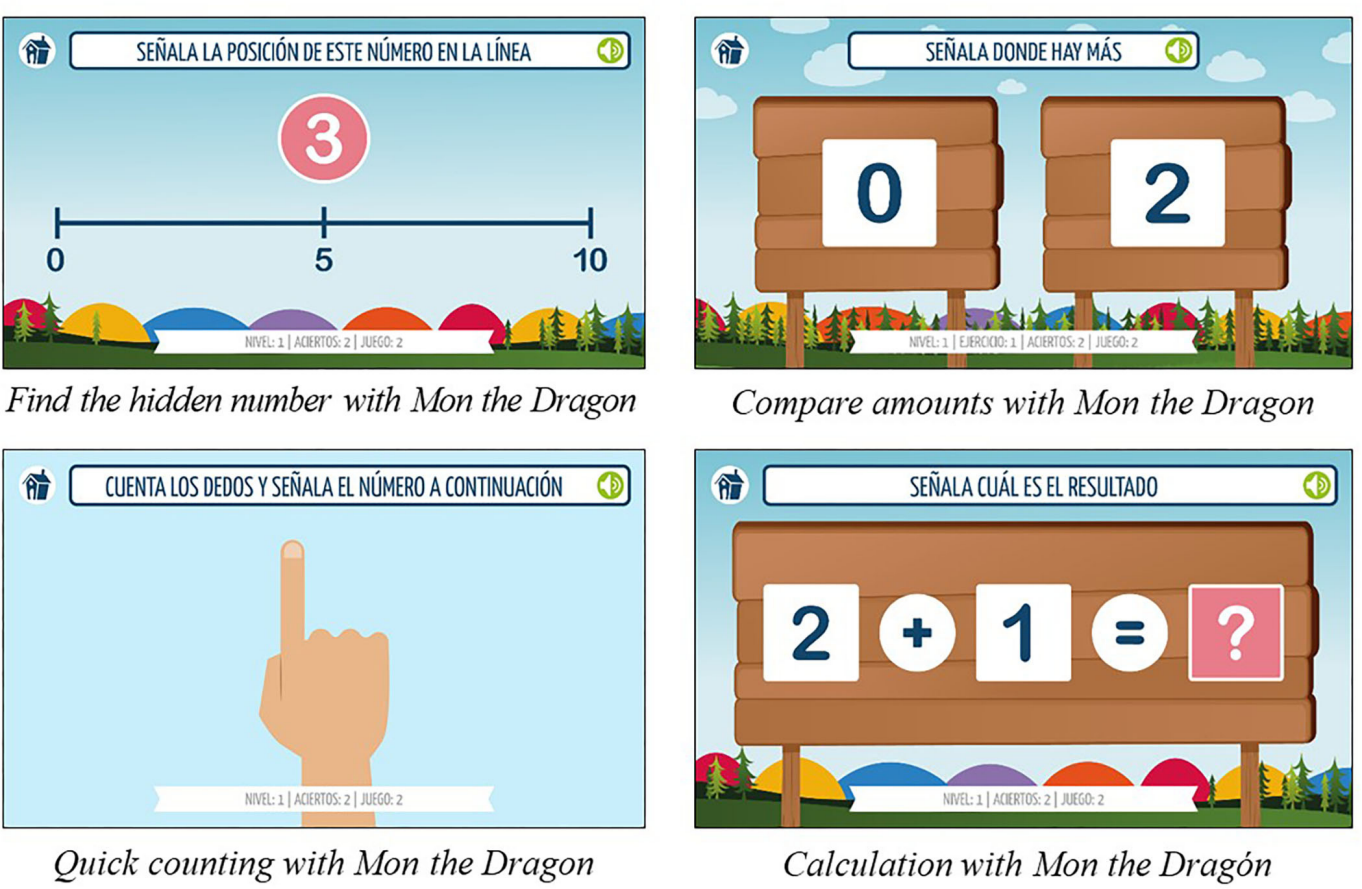
Illustrations of the App designed for the study.

The intervention programme used consisted of 4 apps. These apps for use on touchscreen devices focus on stimulating and training the cognitive foundations associated with early mathematics learning through a simple game in which the participant only has to press a finger on the screen to answer (Mera et al., 2019) (Figure 1). Tablet devices are mobile, lightweight, and do not rely on the motor skills needed to use other technologies, such as a computer keyboard and mouse (Kucirkova et al., 2014).

· *Compare amounts with Mon the dragon*. The game consists of discriminating quantities symbolically and non-symbolically represented, depending on the level of difficulty.
· *Quick counting with Mon the dragon*. The aim of the game is to develop the ability to discriminate small quantities; to count suddenly (without the need to point to each element); and to identify the position of the number within a number line.
· *Calculation with Mon the dragon*. The game aims to develop the ability to perform simple calculations that are stored in long-term memory (addition, subtraction, multiplication, or division), useful in the understanding and development of arithmetic concepts and facilitating problem-solving.
· *Find the hidden number with Mon the dragon*. Two estimation modes coexist in the app: (1) a number is shown and the child must place it in the appropriate position on a straight line (number-position mode); and (2), a mark is shown on the straight line and the player must determine approximately which number would occupy that place (position-number mode).

### Procedure

**Figure 2:**
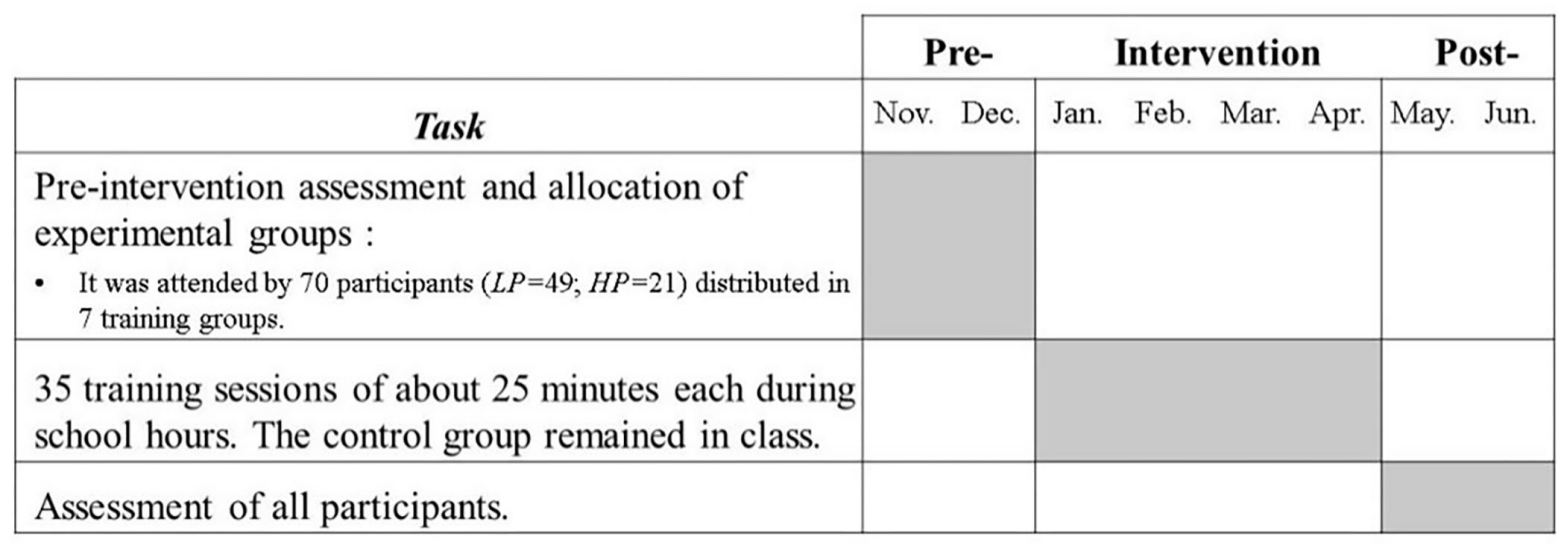
Procedural planning.

After the assignment of the intervention and control groups, 7 working-session groups were generated with 10 participants in each (*N* = 70). The empirical study was conducted using pre- and post-intervention measures. The sequence of the research had three phases, i.e., pre-, intervention and post-intervention. In the intervention phase, each working group carried out 35 sessions of ~25 min. The sessions were carried out in a separate classroom, in good working conditions, and during school hours, respecting break times or non-formal educational activities. During the training of the experimental groups, the control group remained in academic activities. In each working session, there were one or two evaluators with training and experience in dealing with preschoolers and using the app (Figure 2).

Each student was provided with a tablet to develop the intervention sessions. After the intervention period, in the post-intervention evaluation, the evaluators were maintained in order to try to minimise any extraneous variables that might appear due to the effect of the evaluator, leaving a minimum time margin of 6 months between the two evaluation periods, so that recall would not influence the results of the tests.

### Data collection during the intervention phase

The apps run through the Internet and allowed immediately collect the user's general data, date, and task completion timing. Likewise, the design of the app made it possible to generate a file with the user's answers: correct responses, errors, levels of difficulty reached, reaction, and response time for each session. All data were stored in a safe-encrypted database for subsequent statistical calculations.

### Statistical analysis

To analyse differences between the three participating groups in early mathematical competence, a descriptive and inferential analysis of the results was carried out. For the descriptive analysis, measures of central distribution and dispersion were calculated, as well as the gains produced in each of the groups. For the inferential analysis, the *Kruskal-Wallis* test was used due to the size of the sample in the *HP group* (*N* = 21). This test is a non-parametric method with the groups replaced by categories. The *Wilcoxon* test was then used to analyse the differences found between each of the groups, which is based on the differences in the absolute value of the records obtained in the two evaluation phases. Finally, the effect size was calculated by applying Cohen's *d* test, which provides us with a practical significance of the results.

## Results

### Influence of app on mathematical competence

The descriptive statistics obtained in the pre-intervention and post-intervention evaluation phases were analysed, and the gains in the TEMA-3 test scores of each group were calculated (Table 1).

**Table 1:** Means, typical deviations and gainings from the TEMA-3 test between the pre- and post intervention phases.

**TEMA-3**	**Pre-intervention** **M (sd)**	**Post-intervention** **M (sd)**	**Gains** **M**
Control group	21.52 (3.74)	28.26 (5.31)	6.74
LP group	16.53 (2.61)	28.16 (4.99)	11.63
HP group	30.48 (5.52)	42.10 (5.01)	11.62

The *control group* recorded a baseline minimum score of 9 and a maximum score of 32 (*M* = 21.52; *sd* = 3.74) on the test (TEMA-3). The LP group obtained pre-intervention scores with a minimum of 7 and a maximum of 20 (*M* = 16.53; *sd* = 2.61). The *HP group* scored a minimum of 24 and a maximum of 39 (*M* = 30.48; *sd* = 5.52). After the intervention was carried out in the experimental groups, the descriptive statistics of the dependent variable during the post-intervention phase showed the following results: in the *control group* (*M* = 28.26 and *sd* = 5.31), in the *LP group* (*M* = 28.16 and *sd* = 4.99), and the *HP group* (*M* = 42.10 and *sd* = 5.01), respectively. It can be seen that the gains were higher for the groups that received instruction by training with the app.

To test the statistical significance of these gains, the *Kruskal-Wallis* test was used, being an extension of the *Mann-Whitney U*-test for 3 or more groups. The result confirmed that there was a significant difference at 95% (*H* = 50.588, *p* < *0.0*5), with the median between the groups considered to be different. Accordingly, *post-hoc* contrasts were performed using the *Mann-Whitney U*-test for the groups (Table 2).

**Table 2:** Results of *post-hoc* tests in pairs on gains in the mathematical competence test.

	**U of** **Mann-Whitney**	**Std.** **error**	**Desv.** **statistical** **test**	**Sig**.
Control- HP	57.179	13.157	4.346	0.000
Control-LP	60.226	9.413	6.398	0.000
HP -LP	3.048	14.534	0.210	0.834

The results of the pairwise comparisons showed, on the one hand, a significant contrast between the *control* and *HP* groups (*U*_(*C*−*HP*)_=57.179, *p* < *0.0*01) and between the control and LP groups (*U*_(*C*−*LP*)_=60.226, *p* < *0.0*01). On the other hand, no significant differences were found between the gains produced by the *LP* and *HP groups*. In view of the results obtained, the gains were significant at 95% between the *control group* and both experimental groups, with non-significant differences and very similar gains in the contrast between the experimental groups.

To analyse the differences found in each of the groups before and after the intervention, the Wilcoxon test showed significant differences between the evaluations of all the intervening groups as follows: the control group (*Z* = –9.477, *p* < *0.0*5), LP *group* (*Z* = –6.099, *p* < *0.0*5), and HP *group* (*Z* = –4.019, *p* < *0.0*5). To check this increase, an effect size calculation was performed for the different groups (Table 3).

**Table 3:** Effect size on the mathematical competence test (pre/post-intervention).

	**Cohen's d test**	**r**
Control group	1.47	0.59
LP Group	2.92	0.82
HP Group	2.20	0.74

The result of the Cohen's *d* test showed that the effect size produced between the pre-intervention and post-intervention assessments in mathematical competence maintained high values in each of the assumptions, being higher in the experimental groups.

### Influence of app on domain-specific cognitive predictors

**Figure 3:**
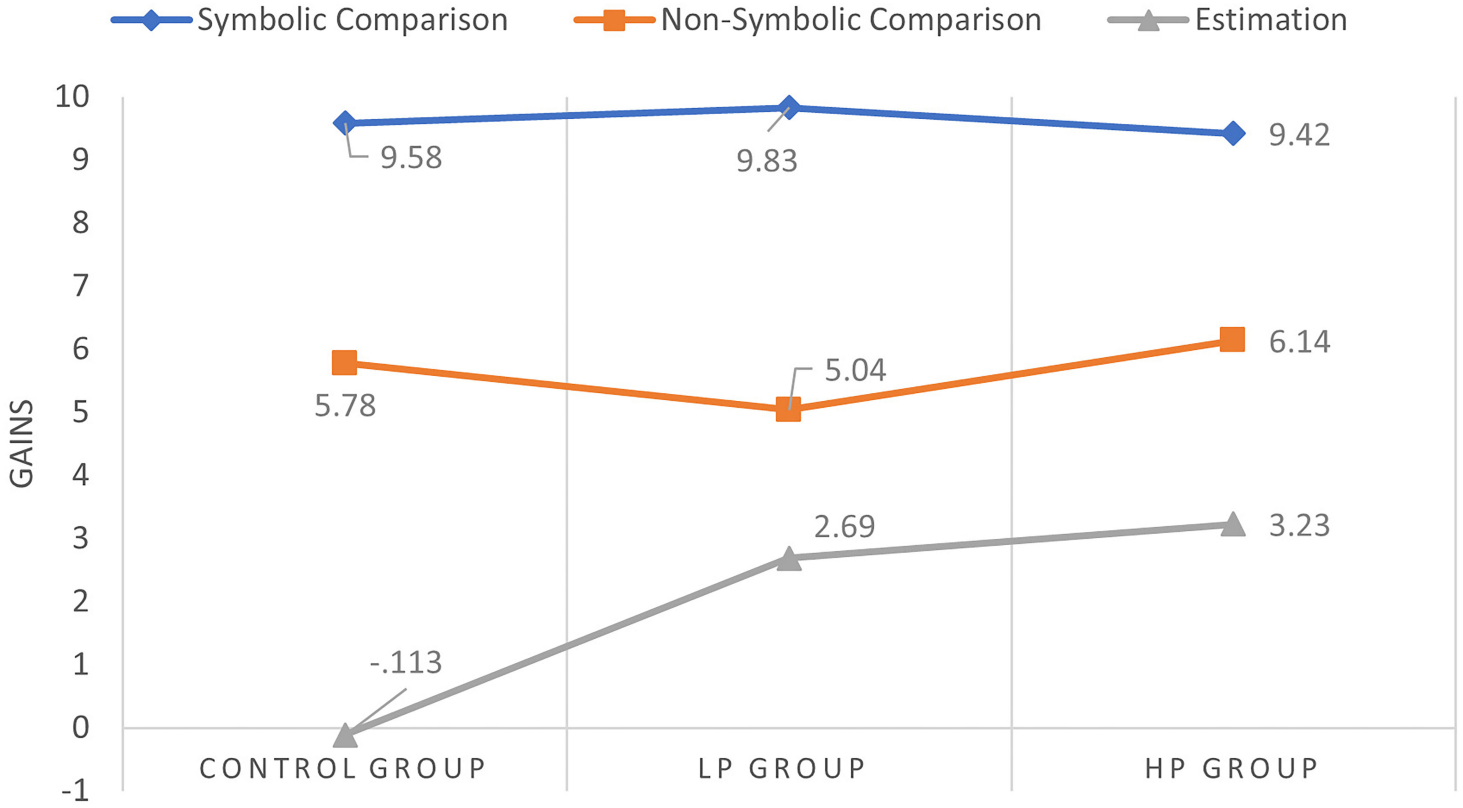
Gains obtained by groups in each of the cognitive predictors assessed after the training programme.

The calculation was to check the influence of using apps over domain-specific cognitive predictors. For the hypothesis testing, descriptive statistics were calculated for the cognitive tests for the assessment of domain-specific cognitive predictors (Table 4) and the increase between groups (Figure 3).

**Table 4:** Descriptive statistics of domain-specific predictors (symbolic and non-symbolic comparison assessed in both assessment phases by groups).

		**Control** **centerM (sd)**	**LP** **M (sd)**	**HP** **M (sd)**
Symbolic comparison	Pre.	31.41 (9.3)	27.12 (8.9)	35.24 (9)
	Post.	41.00 (7.3)	36.96 (8.9)	44.67 (8.4)
Non-symbolic comparison	Pre.	34.19 (6)	32.49 (6.2)	34.38 (7.8)
	Post.	39.97 (6.1)	37.53 (8)	40.52 (5.8)
Estimate	Pre.	5.27 (1.9)	5.20 (1.8)	5.86 (2.1)
	Post.	5.15 (2.1)	7.90 (1.7)	9.1 (0.99)

LP, Low Performance; HP, High Performance.

Respecting the symbolic and non-symbolic comparison, all groups increased in the post-intervention evaluation phase compared to the pre-intervention assessment. In the numerical line estimation task, the *control group* mean in pre-intervention was 5.27 (*sd* = 1.9), while in post-intervention, the result was slightly lower with a mean of 5.15 (*sd* = 2.01). However, in both the *LP* and *HP groups*, scores were higher, increasing scores in both groups.

In the numerical line estimation task, the *control group* mean in pre-intervention was 5.27 (*sd* = 1.9) while in post-intervention the result was slightly lower with a mean of 5.15 (*sd* = 2.01). However, in both the *LP* and *HP groups*, scores were higher, increasing scores in both groups.

In order to test the gaining means differences between the three groups, the *Kruskal-Wallis* test confirmed significant differences in the estimation task at 95% (*H* = 40.983, *p* < *0.0*5). In the symbolic and non-symbolic comparison tasks, the analysis of variance calculation indicated that there was no statistically significant difference.

Contrast tests confirmed significant gaining differences between the control and *LP groups* and between the *control* and *HP groups*. However, no statistically significant differences were found between the *LP* and *HP* groups. In contrast, the largest gains occurred between the control and *HP groups* (Table 5).

**Table 5:** Result of *post-hoc* by couples testing in the estimation task.

	**Mann-Whitney** **U**	**Std.** **error**	**Std.** **desv**.	**Sig**.	**Adjust** **sig**.
Control-HP	59.505	13.120	4.535	0.000	0.000
Control-LP	50.199	9.387	5.348	0.000	0.000
LP–HP	−9.306	14.493	−0.642	0.521	1.00

LP, Low Performance; HP, High Performance.

Considering the variances of each group between the pre- and post-assessment, the *Wilcoxon test* showed significant differences in the estimation task in the pre-intervention and post-intervention evaluations were presented in the *LP* (*Z* = –5.027, *p* < *0.0*5) and *HP* (*Z* = –3.670, *p* < *0.0*5) groups. Regarding the groups that received instruction using the app, the control group (*Z* = −*0.0*35, *p* > *0.0*5) did not show significant differences (*Z* = −*0.0*35, *p* > *0.0*5) between the two assessment phases in the estimation task. In the symbolic comparison task, significant differences were observed for the different groups as follows: *control* (*Z* = –8.268, *p* < *0.0*5), *LP* (*Z* = –5.385, *p* < *0.0*5), and *HP* (*Z* = –3.304, *p*< *0.0*5). In relation to the non-symbolic comparison task, the range comparison between pre-intervention and post-intervention showed a significant difference in each of the groups as follows: *control* (*Z* = −7.020, *p* < *0.0*5), *LP* (*Z* = 3.936, *p* < *0.0*5), and *HP* (*Z* = 3.287, *p* < *0.0*5).

The effect size was high in all groups in both the symbolic and non-symbolic comparison tasks. As Table 6 shows, statistically significant differences were only observed in the estimation tasks in both experimental groups. In this sense, a larger effect size was found in the *HP group* followed by the *LP group* as a result of Cohen's *d* test. However, the control group showed no changes between the two evaluation phases.

**Table 6:** Results of Cohen's d test to calculate the effect size between pre- and post-intervention for the symbolic and non-symbolic comparison tasks.

**PRE/POS–intervention**		**Control** **Group**	**LP** **Group**	**HP** **Group**
Comparación Simbólica	Cohen's d test	1.147	1.105	1.083
	R	0.497	0.483	0.476
Comparación No-Simbólica	Cohen's d test	0.955	0.704	0.893
	R	0.431	0.332	0.407
Estimación	Cohen's d test	−0.05	1.54	1.97
	R	−0.02	0.61	0.7

LP, Low Performance; HP, High Performance.

## Discussion

Currently, part of the scientific community is focusing on the use of technologies and their influence on the development of young learners (Hatzigianni and Kalaitzidis, 2018). Easy-to-access devices are generally used as entertainment tools. Paediatricians, psychologists, and educators advise careful monitoring and limiting the use of digital devices among young children (Kabali et al., 2015).

The scientific literature focused on addressing the development of early mathematical cognition sustains the meaning of training different skills as a cognitive basis for learning math (Geary et al., 2013; Aragón et al., 2014, 2020; Soto-Calvo et al., 2015; Malone et al., 2019; Pace et al., 2019). These skills also provide the development of a solid background in early numeracy, which is considered critically important for later mathematical achievement (Ramani et al., 2017).

The mental number line is fundamental for learning mathematics. In fact, the basis of numerical cognition is considered an innate representation of numerical magnitude in the form of a numerical mental line (Dehaene, 1997, 2003). Recent studies consider that the specific-domain cognitive task of mathematics of estimating a numerical value and its positioning on a straight line has a high predictive value for mathematical achievement (Zhu et al., 2017; Cerda et al., 2018; Schneider et al., 2018; Núñez-Peña et al., 2019).

As a result of this study, the use of the app provided a statistically significant improvement in numeracy skills in both groups compared to the control group. Data suggest that these technological tools can be used for teaching math and helping early childhood educators to provide new experiences for their students (Mattoon et al., 2015).

Apps have also verified their effectiveness in mathematics learning, offering individualised instruction and using technological tools to promote this improvement (Schacter and Jo, 2017; Miller, 2018; Outhwaite et al., 2019; Schenke et al., 2020). However, some studies have found no significant intervention effects with the use of programmes that can be purchased from the current digital platforms (Hellstrand et al., 2020). The critical issue is that it is necessary to experimentally prove its effectiveness in educational settings. Such an evaluation has been possible in this study. App training contributed to improving the mathematical performance of 5-year-old students. The app had educational usefulness.

In relation to the influence of the app on the mathematical learning cognitive background, the number line estimation task showed a higher gain in groups that had received the instruction program.

Positive results were achieved with both types of estimation tested, i.e., number-position and position-number. Estimation is considered critical for learning mathematics. Numerical cognition could be attributed to the representation of numerical magnitude in the form of a mental line and spatial-numerical associations that are already active in early childhood (McCrink and De Hevia, 2018).

Second, both low and high achievers experienced a statistically significant increase in performance in estimation. These results suggest that the instructional system is adaptable and usable by any new mathematics learner, regardless of starting level. These data are more relevant considering that other studies have suggested the causal role of these skills in mathematics (Obersteiner et al., 2013; Moeller et al., 2015; Rugani et al., 2015).

Third, the results contrast with the control group performance, whose average scores in the post-assessment decreased. This suggests that number line estimation tasks could be included in the educational itinerary of early education students. These data are consistent with several contributions. In fact, a meta-analysis (Schneider et al., 2018) with a total of 10,576 participants (aged 4–14 years) showed that the ability to estimate on the number line was a robust tool for diagnosing and predicting numeracy, increasing with age.

As for the magnitude comparison, the learning gainings in the different instructional groups were similar. No significant differences were observed between the groups that had received the instructional programme and the control group. These results could be explained by the accessibility of this type of task for students in the second cycle of early education. The official educational curriculum in Spain and other countries establishes the need to accurately estimate collections or quantities of continuous subjects. Despite not finding statistically significant differences in the use of the app, in terms of magnitude comparison, this task should be relevant to be taught through this instructional programme due to its potential importance in improving mathematics skills (Laski and Siegler, 2007; De Smedt et al., 2009; Matejko and Ansari, 2016; Xenidou-Dervou et al., 2017; Cueli et al., 2019a). Comparing quantities development becomes relevant for improving mathematics learning as this task is part of the ANS for non-symbolic comparison and the PNS for symbolic comparison. Both are closely related to estimation on the number line (Siegler and Booth, 2004; De Hevia, 2016; Reynvoet et al., 2016; Zhu et al., 2017). One example of this is that symbolic comparison and, to a lesser extent, number line estimation skills of early childhood students are predictive of numeracy and longitudinal predictors of overall mathematics performance at these ages (Toll et al., 2015; Lourenco and Bonny, 2017; Mera et al., 2017; Hawes et al., 2019).

Symbolic and non-symbolic magnitude processing skills show different developmental trajectories, with symbolic skills showing greater gains than non-symbolic skills during the early educational stages (Matejko and Ansari, 2016). Similar results were found in this study. A possible explanation for these results may also be related to the type of test used for the assessment of ANS, as results in favour of training are found in the estimation test (van ‘t Noordende et al., 2021), but not in the non-symbolic comparison test. This fact does not call into question the involvement of the ANS in the development of mathematical competence, but these results may be attributable to the measures used.

This is a research study sustaining the trajectory of the last decade of research in educational psychology. It emphasises how important it is to analyse cognitive variables linked to mathematical learning from the first schooling years. The research focuses on the use of technological contributions to the field of education as contrasted tools. These tools can be used rationally in both formal and informal mathematics education. The study has the limitations of quasi-experimental studies. In choosing the experimental sample, we do not have truly random matched groups (which are always difficult to select in educational and clinical settings). For future research, a similar design could be carried out but within an experimental study analysing the influence of the independent variables without the bias of learning difficulty or optimal performance, as has been the case in this study. Considering another concern about the study, we refer to the potential influence that the general predictors could have on the specific ones. That is, there are no purely specific predictors since to carry out any specific mathematical skill, general skills are also required (perceptive, executive, attentional, and processing speed).

## Data availability statement

The raw data supporting the conclusions of this article will be made available by the authors, without undue reservation.

## Ethics statement

The study was reviewed and approved by the Ethics committee of the Research in Cádiz. The legal protocols of ethical guarantees for the development of the study were followed at all times, both in relation to authorizations and data protection. This work followed the International Code of Ethics in the Humanities and Social Sciences of the Centre for Research Ethics and Bioethics. Written informed consent was obtained fromthe parents or guardians of the participants, as well as from the schools.

## Author contributions

CM: application of assessment tools and intervention programme in educational settings, statistical analysis of results, and theoretical discussion of early mathematical competencies. CD: literature review, application of assessment tools in educational settings, and writing the final version of the manuscript. EA: students assessment, statistical analysis, theoretical discussion of mathematical cognition, and contextualisation of statistical results according to the scientific literature. IM: references updating, translation, and reviewing the different manuscript versions. MC: application of assessment tools and intervention programme in educational settings. JN: students assessment, statistical analysis, theoretical discussion of psychometric instruments applied, analysis of comparable results in literature and review of the different manuscript versions, and in charge of the ethical consent of participants. All authors contributed to the article and approved the submitted version.

## Funding

This study was supported by the MINECO/FEDER Spanish Government grants (PID2019-105584GB-I00 MINECO/FEDER and PID2020-119561RB-I00).

## Conflict of interest

The authors declare that the research was conducted in the absence of any commercial or financial relationships that could be construed as a potential conflict of interest.

## Publisher's note

All claims expressed in this article are solely those of the authors and do not necessarily represent those of their affiliated organizations, or those of the publisher, the editors and the reviewers. Any product that may be evaluated in this article, or claim that may be made by its manufacturer, is not guaranteed or endorsed by the publisher.
